# What Can Resting-State fMRI Data Analysis Explain about the Functional Brain Connectivity in Glioma Patients?

**DOI:** 10.3390/tomography8010021

**Published:** 2022-01-27

**Authors:** Giovanni Sighinolfi, Micaela Mitolo, Claudia Testa, Matteo Martinoni, Stefania Evangelisti, Magali Jane Rochat, Matteo Zoli, Diego Mazzatenta, Raffaele Lodi, Caterina Tonon

**Affiliations:** 1 Department of Biomedical and Neuromotor Sciences, University of Bologna, 40138 Bologna, Italy; giovanni.sighinolfi3@unibo.it (G.S.); stefani.evangelisti4@unibo.it (S.E.); matteo.zoli4@unibo.it (M.Z.); diego.mazzatenta@unibo.it (D.M.); raffaele.lodi@unibo.it (R.L.); 2 Functional and Molecular Neuroimaging Unit, IRCCS Istituto delle Scienze Neurologiche di Bologna, 40139 Bologna, Italy; micaela.mitolo@unibo.it (M.M.); claudia.testa@unibo.it (C.T.); magalijane.rochat@unibo.it (M.J.R.); 3 Department of Experimental, Diagnostic and Specialty Medicine, University of Bologna, 40138 Bologna, Italy; 4 Department of Physics and Astronomy, University of Bologna, 40127 Bologna, Italy; 5 Neurosurgery Unit, IRCCS Istituto delle Scienze Neurologiche di Bologna, 40139 Bologna, Italy; m.martinoni@isnb.it; 6 Pituitary Unit, IRCCS Istituto delle Scienze Neurologiche di Bologna, 40139 Bologna, Italy; 7 IRCCS Istituto delle Scienze Neurologiche di Bologna, 40139 Bologna, Italy

**Keywords:** resting-state fMRI, glioma, functional connectivity, network localization, longitudinal study

## Abstract

Resting-state functional MRI has been increasingly implemented in imaging protocols for the study of functional connectivity in glioma patients as a sequence able to capture the activity of brain networks and to investigate their properties without requiring the patients’ cooperation. The present review aims at describing the most recent results obtained through the analysis of resting-state fMRI data in different contexts of interest for brain gliomas: the identification and localization of functional networks, the characterization of altered functional connectivity, and the evaluation of functional plasticity in relation to the resection of the glioma. An analysis of the literature showed that significant and promising results could be achieved through this technique in all the aspects under investigation. Nevertheless, there is room for improvement, especially in terms of stability and generalizability of the outcomes. Further research should be conducted on homogeneous samples of glioma patients and at fixed time points to reduce the considerable variability in the results obtained across and within studies. Future works should also aim at establishing robust metrics for the assessment of the disruption of functional connectivity and its recovery at the single-subject level.

## 1. Introduction

In recent decades, functional magnetic resonance imaging (fMRI) has strongly developed as a non-invasive tool for studying the human brain’s functioning and how different regions of the brain interact and connect. The analysis of functional brain activity through fMRI is achieved by measuring the fluctuations of the blood-oxygenation-level-dependent (BOLD) signal, which can be performed via two main approaches [1]: task-based fMRI (tb-fMRI), where the activation of specific brain circuits is triggered by the execution of a certain task by the patient, and resting-state fMRI (rs-fMRI), where the patient is instead instructed to lie still, without thinking about anything specific. These techniques aim to explore the properties of functional connectivity (FC) within the brain, defined as the temporal correlation between the low-frequency BOLD fluctuations of distinct brain regions [2].

Currently, one of the main applications of fMRI regards the evaluation of FC in presurgical patients with brain tumors, with the primary goal of localizing functionally active regions that must be preserved during the following surgery, yet optimizing the resection. Tb-fMRI is already widely applied for this purpose, as it was found to be reliable in identifying the motor and language networks [3,4]. Moreover, meta-analytic studies and comparisons with direct electrical stimulation (DES), the gold standard in terms of localization of functional areas at the intraoperative level, support that tb-fMRI is a robust tool for presurgical motor planning [5] and may mitigate morbidity and neurological deficits in the postoperative phase [6]. Nevertheless, this technique presents some limitations, mainly associated with the acquisition: it is necessary for the patients to be able and suitable to be trained to perform the task; therefore, they should not present severe neurological deficits or cognitive impairment. Moreover, specific medical and MR-compatible equipment is needed for executing certain tasks, such as keyboard, headphones, or goggles, which in turn require training the technical personnel for the instrumental set-up and the design of the paradigm. Finally, it should be taken into account that to explore the involvement of all brain areas potentially involved by the lesion, several tasks are often needed, thus causing an increased duration of the MR exam.

The application of rs-fMRI in the clinical routine is acquiring increased interest [7,8], especially in the surgical practice, which appears to be a promising field [9]. Using rs-fMRI in this context can be particularly useful, as it would allow overcoming some limitations related to the acquisition of the task-based fMRI sequence, as previously described. Firstly, in most cases, the duration of the MR exam would be reduced, as a single sequence (typically lasting about 8–10 min) can be used to reconstruct different networks (possibly more than the ones localized through tb-fMRI). Even though the possibility of tb-fMRI replacement is still debated [10], mostly because of the intrinsic difference between tb- and rs-derived functional maps, recent reviews in this field have been reporting that network localization through rs-fMRI can be feasible [11,12]. The main reason why the use of this technique is not routinely implemented lies in the complexity and heterogeneity of resting-state analysis: a standardized and robust procedure to recursively identify functional networks has yet to be established [7] and several methods exist [13,14]. The two standard approaches for this purpose are seed-based analysis (SBA) and independent component analysis (ICA). The former retrieves FC by correlating the temporal fluctuations of the BOLD signal of a specific area of interest, chosen with an a priori hypothesis, with all the other voxels in the brain; the latter is a data-driven method, based on the hypothesis that the fMRI data are separable into a set of spatial and temporal statistically independent sources of signal and noise, and thus decomposes the data with the aim of maximizing this independence, directly isolating entire networks of FC and noise components. An additional approach, for which the most recent results are described in this review, is the adoption of deep learning techniques, neural networks (NN) in particular, trained for the reconstruction of the functional networks. From the technical perspective, the vast majority of studies reviewed in this work used a 3T scanner for the acquisition of the images, which constitutes the current standard for research purposes, with a Gradient Echo—Echo Planar Imaging sequence and an intermediate temporal resolution (TR ~ 2 s). The pre-processing pipeline was very similar across studies, and the most common steps (head motion correction, spatial smoothing, temporal filtering, registration to structural images and to standard space, noise reduction) were shared by each one [15]. The analysis as well was generally performed using common software and tools, such as FSL [16], SPM [17], and their toolboxes.

A further advantage related to the use of rs-fMRI is the possibility to deepen the study of the brain FC by performing more specific analysis, with the aim of assessing the functional reorganization of brain connectivity related to the clinical characteristics of the tumor. A paradigmatic example of such techniques is graph analysis (GA) [13,18], which consists of the application of the graph theory to the brain connectivity: the brain is represented as a set of nodes (typically corresponding to Regions Of Interest, ROIs) interconnected through links, which describe the functional connectivity among them. The graph theory gives access to a wide variety of measures whose ultimate goal is to describe the segregation and integration properties of the brain network, i.e., how efficient the communication within clusters of functionally close nodes is and how strongly these clusters are connected among each other [19]. Statistically significant alterations of these features, FC, or other FC-derived metrics, emerging from the comparison with a group of healthy controls (HCs) (or a network known to be unaltered by the lesion) may, in fact, reveal the existence of potential biomarkers of the condition of the patient or the developmental state of the tumor [20,21]. Moreover, the same analytical approach can be applied to study the longitudinal evolution of the brain plasticity consequent to the resection of the tumor, possibly at different time points. These evaluations have not been outlined in previous reviews [11,12,20,21]. Such an aspect would be fundamental for the patient’s quality of life, as it would allow the observation of the functional recovery of the brain, thus the preservation of cognitive and motor abilities. In addition, exploring the capabilities of functional reorganization of the brain following the removal of a tumor and identifying potential biomarkers to predict the prognosis after surgery would be greatly valuable from a scientific point of view.

**Figure 1:**
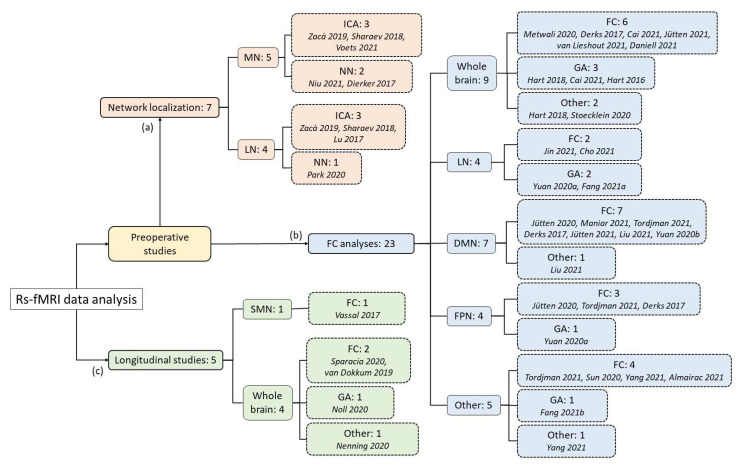
Diagram representing the number of papers reviewed for the present work, subdivided on the basis of their goal, functional network studied, and analytical method used, reaching a total of 1452 patients examined [22,23,24,25,26,27,28,29,30,31,32,33,34,35,36,37,38,39,40,41,42,43,44,45,46,47,48,49,50,51,52,53,54,55]. The references are reported in order of citation. Certain studies explored different aspects; therefore, the sum of subgroups may be greater than the actual number of articles. MN: motor network; LN: language network; DMN: Default Mode Network; FPN: fronto-parietal network, SMN: sensorimotor network; ICA: independent component analysis; NN: Neural Network; FC: Functional Connectivity evaluation; GA: graph analysis.

The purpose of this review was to summarize the results of the most recent studies reported in the literature that used the resting-state fMRI technique in patients with brain glioma, with the aim of (a) localizing and identifying the functional networks in the presence of tumor; (b) characterizing the organization of FC in the preoperative stage; (c) longitudinally characterizing the reorganization of FC after surgery. A diagram of the studies considered can be visualized in Figure 1. Further details on all studies are available in Supplementary Table S1.

## 2. Preoperative Evaluations

### 2.1. Network Localization

The latest implementations of rs-fMRI analysis for the localization of the areas involved in functional networks are mainly performed via ICA and NN, which were also used in the studies reviewed here. The applications for this purpose, in fact, also have as a secondary objective to automatize the identification process, typically by using a matching template procedure. This goal is not flawlessly achievable using SBA, since the latter requires that the pre-selected region chosen as a seed is not directly involved by the tumor location; therefore, the above-mentioned techniques are currently preferred.

Studies mainly focus on the localization of the motor network (MN) and the language network (LN), as these are the ones used in the tb-fMRI approach, which, together with acknowledged templates of the networks, is often taken as a comparison for assessing the quality of the reconstructions. Moreover, cognitive and motor abilities are the most important ones to be preserved in order to ensure the patient’s quality of life after surgery. However, rs-fMRI has the capability of isolating other functional networks [13], even at the single-subject level, as demonstrated by Zacà and colleagues [22], who developed a toolbox for the automatic detection of the MN, LN, and visual (VN) networks. Despite the low number of patients tested, the authors reported a high spatial correspondence (~80–100%) with intraoperative DES.

#### 2.1.1. Motor Network

**Figure 2:**
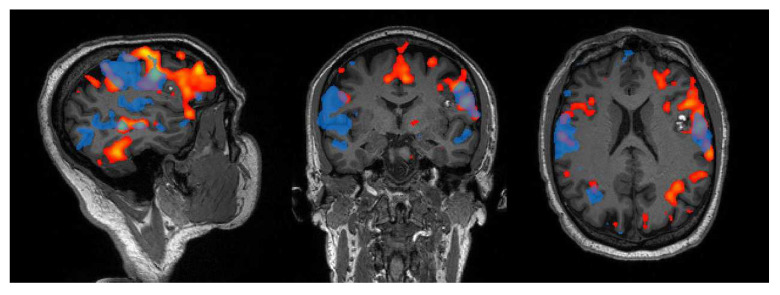
Example of the language network retrieved from a patient with left inferior frontal cavernoma, using a phonemic fluency task (red) and resting-state (blue) fMRI. For the latter, a 10 min-long multiband resting-state fMRI sequence with TR = 0.735 was acquired on a 3T Siemens Magnetom Skyra scanner (IRCCS Istituto delle Scienze Neurologiche di Bologna), and the network localization was performed using ICA with dimension = 30. With respect to task, the resting-state fMRI network exhibits wider activations on the contralateral side (right), yet less extended on the lesioned one (left), especially in the frontal area.

Using ICA, the MN has been successfully identified in patients affected by supratentorial glioma in different recent studies [23,24]. However, Voets and colleagues [24] reported that this approach was not able to isolate the MN in every patient, failing in ~14% of the cases. Despite this, it was also shown that fast multiband sequences, using a TR lower than 1 s, ensure a considerably higher success rate (97.9%) in terms of localization and are therefore advisable for this purpose (see Figure 2 as an example of networks retrieval with a similar technique). Supporting this statement, they also found that the same sequence achieved better concordance both with the tb-fMRI maps and the motor cortex template. This is particularly significant considering that other studies using longer TR [23] only report discrete overlap between the two fMRI approaches (Dice coefficient ~0.3). It must be noted that constrained versions of ICA, where a priori spatial information concerning the regions known to be functionally involved in the network are fed to the algorithm, show improved concordance with tb-fMRI [23].

When adopting deep learning approaches, even better concordance with tb-fMRI can be achieved. In a study comparing NN- and ICA-derived motor maps with the ones retrieved from tb-fMRI [25], it was shown that the former had significantly higher overlap than the latter with task-based maps, reaching a Dice coefficient of 0.39–0.51 vs. 0.33–0.45 (depending, respectively, on whether the hemisphere without or with the glioma was considered). Nevertheless, it must also be noted that the NN itself was trained to derive tb-like maps from the rs-fMRI data. Moreover, it was also found that head motion significantly worsened the correlation with the task-based results, thus suggesting that the removal of artifacts due to patients’ movement from the images can improve the outcomes. Another deep-learning approach, multi-layer perceptron (MLP), trained to assign each voxel to one of seven RSNs, was used to demonstrate that the MN derived from rs-fMRI exhibited higher overlap with the motor regions defined by FreeSurfer and Broadmann areas than the tb-fMRI ones [26].

#### 2.1.2. Language Network

Using a discriminability-index-based component identification algorithm for the automatic recognition of the LN among the ICA components, Lu and colleagues [27] were able to achieve significant results in terms of automatic identification of the network in healthy controls and glioma patients and high sensitivity in the overlap of the reconstructed maps with the DES points: 87% when considering a 1 cm radius from the stimulated region against 47.8% obtained through SBA. Another study using both ICA and semi-blind ICA [23] to identify the LN, despite exhibiting more variable results depending on the infiltration of the tumor in the language areas, showed even higher concordance than the motor one with the corresponding tb-fMRI activation outcomes (Dice coefficient ~0.5 when language areas were not directly involved by the lesion and ~0.2 otherwise). 

The same MLP method described for the reconstruction of the MN was also used to reconstruct the language one [28]. Compared to predefined LN templates, the rs-fMRI data achieved optimal overlap with Wernicke areas, better than tb-fMRI, whereas in Broca areas, the trend was inverted. However, the rs-fMRI data showed good concordance with the tb-fMRI result, as the peaks of functional activation for both methods were localized in Broca- and Wernicke-like areas.

To summarize, promising results have been achieved lately in the identification and localization of the MN and the LN in glioma patients, making rs-fMRI a valid alternative to tb-fMRI when the latter cannot be performed. In particular, the first applications of deep learning methods have been showing that such an approach can achieve excellent outcomes for the specific purpose of reconstructing tb-fMRI-like networks, starting from the rs-fMRI data. Nevertheless, it is also significant to note that ICA is already widely acknowledged, so much so that it is also used to isolate the functional networks in studies that do not focus on their identification but rather aim at characterizing their properties in terms of FC [29,30,31,32].

Moreover, the presence of gliomas clearly influences the localization of a functional network [11], not only because the regions involved by the lesion cannot be activated but also due to brain functional reorganization mechanisms [20,21], as detailed in Section 2.2. Nevertheless, the good concordance between the results achieved using rs- and tb-fMRI data ensures that such alterations can also be captured using the resting-state technique. For instance, Voets and colleagues [24] evaluated the overlap between the sensory-motor network identified using tb- and rs-fMRI approaches in a cohort of healthy controls and glioma patients, demonstrating that the overlap is significantly consistent in both groups.

### 2.2. Functional Connectivity Analysis

Recent findings regarding the functional reorganization of the brain related to the presence of a glioma have shown that FC is significantly altered not only in regions close to the tumor but also at a considerable spatial distance and at the whole-brain level. In fact, significant differences compared to healthy controls were detected between entire functional networks (such as between the default mode network and the dorsal attention network with the fronto-parietal network) and with the cerebellum [33]. Even at lower spatial scales, i.e., considering ROIs rather than full networks, it was observed that the distribution of the FC values among brain regions differed significantly between patients and controls, being broader, with higher peak position and smaller peak height in the former group [34]. Another interesting example is reported by Hart and colleagues [35], who used fractal analysis to explore the complexity of the BOLD fluctuations, with the hypothesis that simple trends in the time series were indicative of ill-conditioned function: they found that the fractal complexity followed a quadratic trend, with low values in the proximity of the tumor, then growing to a peak at about 5 cm from the tumor center, and finally going back to values comparable to those of HCs at ~9 cm. This can be interpreted as a compensatory mechanism mediated by regions at an intermediate distance from the glioma due to the reduced activity in the proximity of the tumor. A newly introduced measure, the ABnormality Index (ABI) [36], which measures how different the connectivity strength of voxels in one hemisphere is compared to the corresponding location in a group of HCs, was proven to be significantly altered in both the tumor side and the contralateral hemispheres (even though prominently in the former). This may indicate that spatially distant areas can indeed be affected, likely because of tumoral cell invasion. 

The FC alterations are also related to other physical or biological characteristics of the tumor, such as the location, the grade, or the isocitrate dehydrogenase (IDH) gene mutation. Grade II gliomas exhibit a higher peak position in the distribution profile of FC than grades III and IV [34], indicating that FC tends to be stronger in the former, and the ABI positively correlates with the tumor grade [36]. It was also proven that FC is a good predictor of tumor grade, achieving ROC area under the curve between 0.85 and 1 in classifying low-grade vs. high-grade gliomas in the left and the right side, respectively [37]. Moreover, patients with wildtype IDH display reduced FC [38] and greater ABI [36] versus controls at the whole-brain level.

FC characterization may also be meaningful for clinical evaluations. Not only is FC able to differentiate between positive and negative DES points [39], providing indications about specific functional locations prior to the surgery, but also new insights may be revealed using GA: functional brain graphs were demonstrated to be robust to random attacks, i.e., the removal of a random node from the network, but very vulnerable to targeted attacks to network hubs, i.e., nodes that have a central role in the communication within the graph [40]. GA could therefore inform the neurosurgeon about the areas that must be preserved to maintain the network (or subnetwork) integrity. In terms of diagnosis and prognosis, the ABI correlated with cognitive performances, the Montreal Cognitive Assessment (MOCA) in particular [36], and a broader FC distribution profile was associated with shorter progression-free survival [34]. Moreover, Daniel and colleagues [41] found that mean intratumor FC in glioblastoma patients may persist despite the presence of the lesion, even with large variability, and this quantity is positively correlated to longer survival.

Concerning additional demographic and clinical characteristics of patients, the handedness was measured in the majority of studies and frequently used as a covariate when homogeneous samples were not available; additionally, neuropsychological and cognitive scores were assessed in a minority of studies, but with an increasing trend in the most recent research. Such information may be fundamental in future studies, as it would allow interpreting the functional reorganization of the brain in the light of the patient’s cognitive status.

Besides the whole-brain findings reported above, several studies have been focusing completely or partly on specific functional networks, which are therefore analyzed in the following paragraphs.

#### 2.2.1. Language Network

The language network is reported to undergo an overall reduction in the FC [29] and network strength [42] in patients affected by gliomas, which was also observed to be associated with the tumor grade [42]. Interestingly, such alterations showed differences based on whether the lesion was located in the left or the right hemisphere: in the former case, the FC reduction emerged within both the hemispheres bilaterally [29], in particular between the Broca and Wernicke areas [43], whereas in the latter case, significantly lower values were observed only in the ipsilateral hemisphere [29]. In contrast to this, another research study, focusing on temporal gliomas, found that the LN only showed lower integration and higher segregation properties in the case of right-sided tumors, whereas those in the left mainly affected the visual network (VN) [44,56]. Moreover, whether gliomas have an impact on the FC between the cerebrum and cerebellar regions remains unclear, as results are conflicting [42,43].

As observed globally, also for the specific case of the LN alterations, correlations with the characteristics of the lesion emerged. Results support that the tumor volume negatively correlates with the ipsilateral interhemispheric and global FC of the LN when the glioma is located in the left hemisphere and positively with the contralateral intrahemispheric FC for right-sided tumors in right-handed patients [29], suggesting that compensation mechanisms of the LN are favored when gliomas are located in the right hemisphere.

#### 2.2.2. Default Mode Network

Regarding the default mode network (DMN), the results reported in the literature are unable to provide a clear and unequivocal picture of the alterations. In fact, from recent studies, both decrement [31] and increment of the overall FC within such network [32], and with the other brain networks [34], were detected. However, it is to note that even where a global increase was found, local negative alterations in the posterior cingulate cortex (PCC) and the precuneus were observed [32]. Similarly, while some studies described a loss of FC in the ipsilateral hemisphere [31], others found the quantity increased in the lesioned side, at least in the case of right-sided gliomas [30]. Again, whether the FC increases [30,45] or decreases [46] in the contralateral hemisphere, and especially between the parahippocampal gyrus, the PCC, and the temporal regions, is debated.

Moreover, the actual advantage of increased DMN connectivity in glioma patients is unclear: while a positive correlation of FC was observed with the Mini Mental Status Exam (MMSE) [46], higher FC was also associated with worse attention scores [30]. From the clinical perspective, results are also contrasting in terms of the relationship with the IDH mutation status: Jütten and colleagues [38] reported reduced FC in mutated IDH patients and preserved in wildtype IDH, whereas other studies did not find a relation between the genetic anomaly and DMN connectivity [32].

#### 2.2.3. Fronto-Parietal Network

Similarly to the DMN, for the fronto-parietal network (FPN), the findings are ambiguous: on the one hand, increased FC among PCC, precuneus, and the frontal cortex [32], or within the contralateral hemisphere [30] in patients is reported; on the other hand, globally lower strength, specifically in high-grade gliomas, was observed [46]. Nevertheless, these results may be reconciled by the finding that the connectivity between the DMN and FPN is stronger in glioma patients, who instead display lower FC strength between FPN and the rest of the brain [34].

A study comparing the FC of the DMN and the FPN during rest and working-memory task conditions found that the level of task-evoked reconfiguration in the networks did not differ significantly based on the tumor type [57]. Considering the heterogeneity in the results exhibited by the different studies reported in this review, it might be of interest to investigate whether the resting-state FC alterations caused by different tumor types are answered by corresponding anomalies in task-based data, thus explaining why the task-evoked reconfiguration may remain unaltered [57]. For instance, in the study of Maniar and colleagues [31], both resting-state and task-based fMRI data, using a phonemic fluency paradigm, were acquired, demonstrating high concordance between the two techniques in terms of FC decrease within the DMN.

#### 2.2.4. Other Functional Networks

Few results have been shown in recent literature concerning the preoperative FC alterations of other functional networks in glioma patients. A loss of the bilateral symmetry of the hand-motor network (hand-MN) emerged from a heterogeneous sample of glioma patients, also showing a correlation with the distance of the tumor from the ipsilateral primary motor cortex [47]. Concerning the salience network (SN), average decreased FC was observed and also supported by a disruption of the amplitude of low-frequency fluctuations (ALFF) in all the ROIs of the network (bilateral dorsal anterior cingulate cortex and anterior insula), even with variable outcomes based on the characteristics of the glioma [48]. The dorsal attention network (DAN) appears to be affected by significant alterations, too; higher FC in frontal and occipital regions and lower in the subcallosum and the anterior cingulate cortex were found independently of the tumor characteristics and mutation status [32]. Another interesting aspect is the specific case of insular diffused low-grade gliomas: independently of the side of the tumor, the contralateral insula shows increased FC, especially towards the visual and sensorimotor networks [49], and higher communication efficiency [50] compared to HCs.

**Figure 3:**
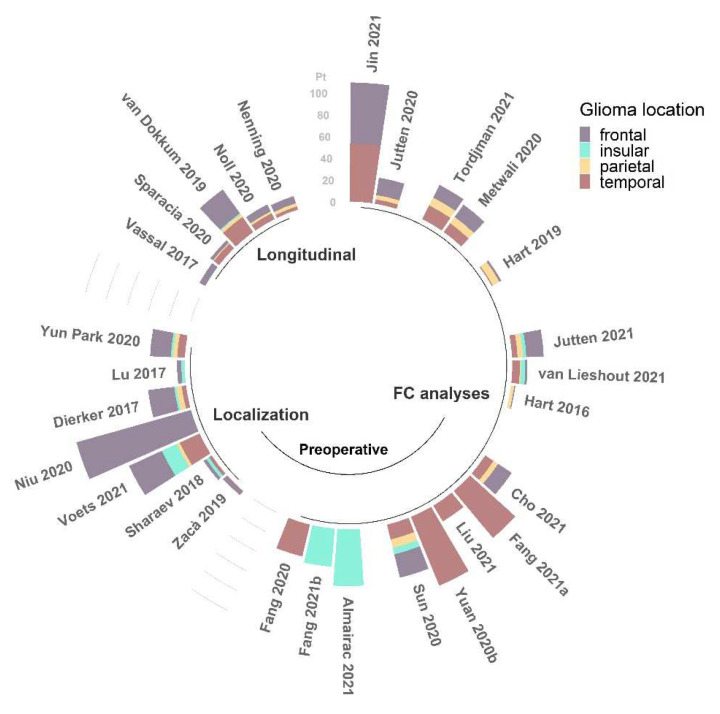
Number of patients (pt) with frontal, insular, parietal, and temporal glioma locations among the studies (N = 28) reviewed for this work [22,23,24,25,26,27,28,29,30,32,33,35,38,39,40,43,44,45,46,47,49,50,51,52,53,54,55,56], grouped by field of application and reported in order of citation. This information was not available for the other 7 studies [31,34,36,37,41,42,48].

**Figure 4:**
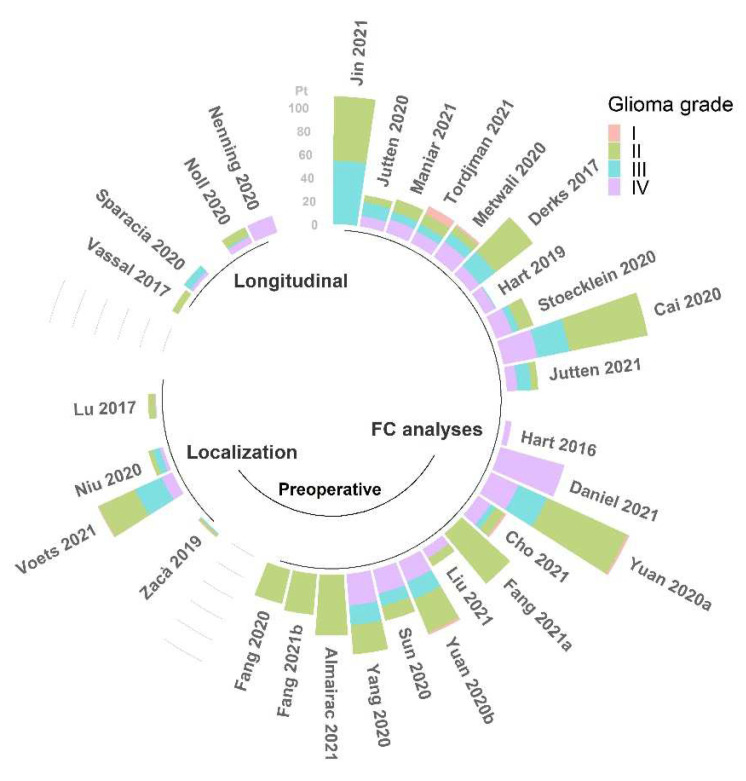
Number of patients (pt) with classified WHO grade glioma among the studies (N = 30) reviewed for this work [22,24,25,27,29,30,31,32,33,34,35,36,37,38,40,41,42,43,44,45,46,47,48,49,50,51,52,54,55,56], grouped by field of application, and reported in order of citation. This information was not available for the other 5 studies [23,26,28,39,53].

In summary, the results reported in the literature concerning alterations of FC, despite providing useful insights about the anomalies caused by the occurrence of a brain glioma, do not achieve complete concordance in terms of the direction of such changes. The main reason for this disagreement is likely related to the considerable heterogeneity of samples, both within and across studies (see Figure 3 and Figure 4). Being able to assess FC properties in more homogeneous groups of patients will improve the power of the rs-fMRI analysis to determine specific alterations associated with different characteristics of the tumors.

## 3. Longitudinal Evaluations

The majority of resting-state fMRI studies in patients with brain glioma only reported presurgical evaluations; however, functional recovery and cognitive outcomes following the tumor resection are of paramount importance. 

Vassal and colleagues in 2017 [51] showed functional changes after surgery at both inter- and intrahemispheric level within the sensorimotor network (SMN), which exhibited an FC reduction 24 h after surgery and significant recovery of connectivity at 3 months of follow-up, which was also associated with the recovery of motor functions. More recently, other authors [52] demonstrated FC changes after surgery in the SMN, but also in the DMN, SN, and LN, even at the single-subject level. Pre- and postoperative alterations of various functional networks were also observed by van Dokkum and colleagues [53], who, before surgery, found FC changes within the SMN, the VN, the ventral DMN, and the visuospatial network. The latter also showed alterations post-surgically, together with the dorsal DMN, the SN, and the post-SN. Moreover, the ventral DMN, the dorsal DMN, and the visuospatial network changes were related to improved picture-naming performances, suggesting the role of the reconfiguration of these networks in patients’ recovery. Besides the FC evaluation, the authors also calculated the “disconnectome score” (ds), i.e., the probability of an ROI derived from a functional network to be anatomically disconnected, based on the number of white matter tracks emanating from or passing through the lesioned area. Using this measure, they observed that after surgery, the average ds of each resting-state network decreased, with the obvious exception of the frontotemporal regions primarily involved by the surgery, implying that the tumor itself caused a disruption that could be partially recovered following the resection. Interestingly, the increased longitudinal disconnectivity did not worsen the language performances obtained by patients compared to the presurgical time point.

In the previously mentioned studies, the follow-up imaging data were acquired at a temporal distance of 1–3 months after surgery. A work by Noll and colleagues [54] focused instead on long-term differences, performing a re-scan after 6 months. The connectomic properties of betweenness centrality and assortativity, evaluated through GA, were significantly smaller postoperatively, and reductions in these measures were associated with better neurocognitive outcomes. Moreover, inverse associations were observed between changes in language, executive functioning, learning and memory performances, and alterations in segregation and centrality network properties.

A last, stimulating work by Nenning and colleagues [55], who were able to re-scan a sub-sample of 6 out of 15 glioblastoma patients for up to 8 bi-monthly follow-ups, introduced a new metric, the anomaly score, which quantifies the similarity of the connectivity matrix of the single patient with the average of HCs. Using this measure, they found a higher anomaly score in voxels functionally close to the tumor, rather than anatomically, especially for high-order, spatially distributed networks (such as DAN and VAN). Additionally, the anomalies were not only present in the tumoral side but also spread symmetrically in the contralateral hemisphere and in the cerebellum, yet typically showing recovery at follow-up. Finally, this metric also appears to have prognostic value since it exhibited an increment two months prior and in spatial correspondence of tumor re-occurrence in 4/5 patients with grey matter tumors. 

From the few longitudinal studies present in the literature, it is clear that brain plasticity following the resection of the tumor impacts the FC. However, it is also evident that the degree of recovery (or alteration) depends on the follow-up time. In this sense, a standardized procedure is lacking, and this can definitely be one of the main sources of variability among studies, which consequently undermines the generalization of outcomes. Further research on this aspect will likely allow obtaining stable and reliable metrics for longitudinal outcomes.

## 4. Conclusions

The present review has shown the capabilities of rs-fMRI data analysis to provide insights on the FC properties of glioma patients.

The identification and localization of different functional networks proved to be feasible, even though not fully comparable to tb-fMRI, and especially the cases of (constrained) ICA in combination with a fast multiband sequence and of deep-learning techniques are worthy of further investigation.

On the other hand, the latest studies reported in the literature do not achieve a full consensus regarding the FC alterations caused by brain gliomas. Even though the disruption of FC is generally reported in the close proximity of the tumor, it remains unclear how the rest of the brain responds to the presence of the lesion. In particular, it is still debated whether gliomas can also be associated with a long-range and cross-hemisphere reduction in FC [36,55] or rather distant regions compensate for the decline by increasing their connectivity with the rest of the brain [35]. As mentioned, the high variability registered among the outcomes is likely due to the considerable heterogeneity in the characteristics of the patients typically examined not only across but also within the studies. The location, grade, type, and mutation status of the glioma are all properties that reasonably influence the functional connectivity features of the brain, as it also emerged from this review: research studies should be focused on more homogeneous samples of patients. Another issue of these studies, which, however, has been scaling down in the most recent works, is the size of the cohort of subjects, especially when separating the latter into subgroups defined by the tumor characteristics, which in turn decreases the statistical power of the outcomes. Overcoming these issues will allow us to speculate more definitely on the relation between the properties of the gliomas and the measured brain FC changes. 

From the longitudinal perspective, a standardized timeline for the evaluation of the FC recovery is lacking. It is, in fact, unclear whether a single temporal point can be suggested to obtain stable results ensuring optimal recovery, as the latter clearly depends on the outcome of the surgery itself, besides other specificities of the patients. The acquisition, where possible, of several temporally interspersed follow-ups would therefore be ideal for tracking changes and achieving consensus on optimal re-scan times, possibly adapted to the type of tumor and the outcome of the resection. Moreover, as shown by some promising results [55], the analysis and comparison of follow-up outcomes may have clinical importance in describing the evolution of the actual condition of patients. 

A relevant perspective for future studies would be to aim at performing single-subject evaluations, which is a topic of great interest for introducing rs-fMRI analysis in the clinical routine [7,8]. Achieving reliable results for this purpose would allow us to reach a personalized characterization of functional connectivity, which in turn would include high diagnostic and prognostic value for patient-specific care. To obtain such results, it is necessary to test the stability of existing and newly introduced measures by verifying their reproducibility on homogeneous samples of patients. Longitudinal studies could provide useful information in this context, as different scans of the same subject are acquired: prior knowledge of regions and networks that should be unaltered at follow-up could be used for testing the actual robustness of the metrics.

Resting-state fMRI data constitute a great source of information, whose analytic complexity also corresponds to the possibility of retrieving a large number of relevant outcomes for clinicians and researchers. Overcoming the sources of variability which characterize studies adopting this technique would ensure the opportunity to consistently explore the existence of functional connectivity alterations retaining diagnostic and prognostic power.

## Data Availability

No new data were created or analyzed in this study. Data sharing is not applicable to this article.

## Supplementary Materials

The following supporting information can be downloaded at: https://www.mdpi.com/article/10.3390/tomography8010021/s1, Table S1: The table includes two different sheets summarizing the studies reviewed for this paper. The first collects the technical aspects, i.e., the acquisition, the analytical methods and the results obtained; the second describes the demographic and clinical characteristics of the populations studied.
